# Differential distribution of linezolid in diseased and nondiseased bones in patients with spinal tuberculosis

**DOI:** 10.1186/s12879-023-08970-x

**Published:** 2024-01-05

**Authors:** Yuan Li, Guohua Lei, Weijie Dong, Tinglong Lan, Jun Fan, Shibing Qin

**Affiliations:** Department of Orthopedics, Beijing Chest Hospital, Capital Medical University, Beijing, China

**Keywords:** Tuberculosis, Spine, Linezolid, Pharmacokinetics

## Abstract

**Background:**

Linezolid exhibits antibacterial activity against sensitive and drug-resistant strains of *Mycobacterium tuberculosis*. Knowledge on the distribution of linezolid in different types of bones in patients with spinal tuberculosis (TB) is lacking, which limits the pharmacokinetic and pharmacodynamic studies of linezolid. This study aimed to evaluate the distribution of linezolid in diseased and nondiseased bones in patients with spinal TB.

**Methods:**

Spinal TB patients treated with linezolid-containing regimens and whose diseased and nondiseased bones were collected during surgery were enrolled retrospectively from January 2017 to February 2022. Blood, nondiseased bones, and diseased bones were collected simultaneously during the operation. Linezolid concentrations in the plasma, nondiseased bones, and diseased bones were subjected to high-performance liquid chromatography–tandem mass spectrometry.

**Results:**

Seven eligible spinal TB patients, including one rifampicin-resistant case, were enrolled. Following a 600 mg oral administration of linezolid before surgery, the median concentrations of linezolid in plasma, nondiseased bone, and diseased bone of the seven patients were 8.23, 1.01, and 2.13 mg/L, respectively. The mean ratios of linezolid concentration in nondiseased bones/plasma, diseased bones/plasma and diseased bones/nondiseased bones reached 0.26, 0.49, and 2.27, respectively. The diseased bones/plasma presented a higher mean ratio of linezolid concentration than nondiseased bones/plasma, and the difference was statistically significant (*t* = 2.55, *p* = 0.025). Pearson’s correlation analysis showed the positively correlation of linezolid concentrations in diseased and nondiseased bones (*r* = 0.810, *p* = 0.027).

**Conclusions:**

Linezolid exhibits a higher concentration distribution in diseased bones than in nondiseased bones.

## Background

Tuberculosis (TB) remains one of the top 10 causes of death and is the leading cause of death from a single infectious agent worldwide [1]. According to the 2022 global TB report, approximately 10.6 million people fell ill with TB in 2021, and 1.6 million individuals died from TB globally. The global cure rate for new and relapse TB is 86%, and that for patients with multidrug-resistant TB (MDR-TB) is 60% [2]. Osteoarticular TB accounts for 1–3% of all TB cases. MDR-TB represents 13.27% (15/113) of patients with osteoarticular TB [3]. Osteoarticular TB often causes bone destruction and dysfunction, which place an enormous burden on families and the society [4].

Linezolid exhibits antibacterial activity against sensitive and drug-resistant strains of *Mycobacterium tuberculosis* [5–6]. Linezolid-containing regimens show potent clinical efficacy in the treatment of sensitive and drug-resistant TB [7–8]. Linezolid achieves a good therapeutic effect in the treatment of TB, but it is accompanied with a high incidence of side reactions [9–10]. This disadvantage limits the clinical application of this compound in the treatment of TB. A previous study showed the heterogeneous distribution of linezolid in spinal TB lesions [11]. Therefore, blood concentration cannot be used to represent the concentration of linezolid in TB lesions.

Linezolid presents different spatial distributions in TB lesions [11–12]. However, knowledge on the differential distribution of linezolid between diseased and non-diseased bones in patients with osteoarticular TB is lacking, which limits its clinical application in the treatment of spinal TB. The present study aimed to evaluate the distribution of linezolid in diseased and nondiseased bones in patients with spinal TB.

## Methods

### Patient categories and enrollment

From January 2017 to February 2022, patients with spinal TB were diagnosed through pathological examination or TB culture (BACTEC MGIT 960) in the Orthopedics Department of Beijing Chest Hospital, Capital Medical University. The patients who were treated with linezolid- containing regimens and had their diseased and nondiseased bone collected during surgery were enrolled retrospectively. Exclusion criteria included (1) allergy to linezolid, (2) surgical contraindications such as severe cardiovascular, liver, kidney, or blood system disease or other serious illnesses, and (3) mental illness. All patients orally took linezolid at a dosage of 600 mg per day before undergoing surgery. On the day of the surgery, they orally received 600 mg linezolid before surgery.

The indication for the administration of a linezolid-containing regimen prior to surgery was the suspected drug-resistant TB in the patients. The indication of suspected drug-resistant TB was failure of first-line anti-TB treatment regimen. After the operation, the anti-TB treatment regimen was adjusted based on the results of Xpert MTB/RIF and drug sensitivity testing. If the patient showed no resistance to rifampicin, then linezolid was discontinued.

### Sample collection and processing

The median period from the administration of the preoperative dose of linezolid in the morning to sample collection during the operation was 150 min (range: 105–220 min). Venous blood, nondiseased bone, and diseased bone samples were collected during the operation. The samples were sent to the laboratory for further processing. Nondiseased bones, which included four spinous processes and three ribs, were the minimum amount of bone that must be removed to prevent the lesion from being exposed and removed during the surgical procedure.

Each venous blood sample was centrifuged at 3000 × *g* for 15 min. Then, the supernatant was collected after centrifugation. The plasma was labeled and stored at − 80 °C until analysis. Attachments were removed first from nondiseased and diseased bone tissues. Each bone tissue sample was weighed and added with ultrapure water at a rate of 1 g/mL. The mixture was homogenized in a FastPrep-24 instrument (MP Biomedicals Europe) for 100 s at 6 m/s by using MP Bio FASTPREP-24. Homogenates were stored at 4 ℃ for 5 h and vortexed every 30 min for drug extraction. The supernatant was collected after centrifugation at 3500 × *g* for 15 min, labeled, and stored at − 80 °C until analysis.

All standard stock solutions were prepared at a concentration of 1 mg/mL. Linezolid was dissolved in methanol and stored at − 70 °C prior to use. Methanol was added to the supernatant separated from venous blood, or nondiseased bone, or diseased bone samples, and mixed thoroughly. After centrifugation for an additional 15 min, the supernatant was transferred to glass injection vials for high-performance liquid chromatography (HPLC) analysis.

### HPLC–tandem mass spectrometry analytical methods

Linezolid concentrations in plasma, nondiseased bone, and diseased bone tissues were analyzed through a validated HPLC–tandem mass spectrometry method using an HPLC system (Agilent Technologies, Santa Clara, CA, USA) equipped with an autosampler (G1329A) and a column heater (G1316A).

We used a G6420A triple quadrupole mass spectrometer (Agilent Technologies) and an electrospray ionization source. The mass spectrometer was operated using the following settings: 5 kV capillary voltage and 22–27 eV collision energy. Quantification was achieved through selected reaction monitoring in positive ion mode. Peak area integration and data analysis were performed using Agilent Mass Hunter software B.08.00 (Agilent Technologies, Santa Clara, CA, USA). Linezolid analysis was performed using a ZORBAX SB-C18 column (2.1 mm × 50 mm; Agilent Technologies). The mobile phase comprised acetonitrile and 0.1% formic acid solution (65:35, v/v) at a flow rate of 0.3 mL/min. The multiple-reaction monitoring transition for linezolid was 338.1→296.1. Calibration curves for linezolid in the range of 0.2–25 µg/mL were established (r^2^ > 0.99).

### Statistical analysis

The continuous variables in this study are presented as the median or mean ± standard deviation (SD). The statistically significant differences in the ratios of linezolid concentrations among different samples/plasms were compared via t tests. The relationship between linezolid concentrations in diseased bone and nondiseased bones was examined using Pearson’s correlation analysis. All tests of significance were two-tailed, and a *p* < 0.05 was considered statistically significant. Analysis was performed using the commercial statistical software SPSS version 13.0 (SPSS Inc., Chicago, IL, USA).

## Results

### Study patients

Seven patients (five males and two females) with spinal TB, including a rifampicin-resistant case, and a median age of 45 years were enrolled. Venous blood, nondiseased bones, and diseased bones were collected from each patient during the operation. The median body mass index (BMI) was 20.76 kg/m^2^ (range: 19.14–25.24 kg/m^2^), and the median linezolid dose by weight was 10.00 mg/kg (range: 9.09–11.54 mg/kg). Table 1 shows the clinical characteristics of the seven studied patients.

**Table 1:** Clinical characteristics of the seven studied patients

Patient No.	Gender	Age (years)	Lesion site	BMI (kg/m^2^)	Dose/Weight (mg/kg)	HIV status	Comorbidity	Anti-TB treatment regimen
1	Female	30	L1-3	21.48	10.52	—^a^	Pulmonary TB	H-Z-Lfx-Pto-Lzd
2	Male	45	T6-7	20.76	10.00	—^a^	Pulmonary TB	H-R-E-Z-Lzd
3	Female	50	T10-11	25.24	9.52	—^a^	— ^b^	H-R-E-Z-Lzd
4	Male	17	L1-2	19.14	9.68	—^a^	— ^b^	H-R-E-Z-Lzd
5	Male	53	T8-9	20.76	10.00	—^a^	Diabetes mellitus	H-R-E-Z-Lzd
6	Male	55	T10-11	20.31	11.54	—^a^	Pulmonary TB	H-R-E-Z-Lzd
7	Male	39	T12-L1	20.37	9.09	—^a^	Pulmonary TB	H-R-E-Z-Lzd
Median	—^b^	45	—^b^	20.76	10.00	—^b^	—^b^	—^b^

No: number; T: thoracic vertebra; L: lumbar vertebra; BMI: body mass index; dose/weight: daily doses

of linezolid/patient’s weight; TB: tuberculosis; HIV: human immunodeficiency virus

^a^Negative

^b^No data

### Linezolid concentrations in nondiseased and diseased bones of spinal TB patients

Following a 600 mg oral administration of linezolid before surgery, the median concentrations of linezolid in plasma, nondiseased bones, and diseased bones of the seven patients measured 8.23 (range: 2.16–12.03 mg/L), 1.01 (range: 0.63–4.05 mg/L), and 2.13 (range: 1.63–5.39 mg/L) mg/L, respectively. The mean ratios of linezolid concentration in nondiseased bones/plasma, diseased bones/plasma, and diseased bones/nondiseased bones reached 0.26 (range: 0.07–0.44), 0.49 (range: 0.25–0.75), and 2.27 (range: 1.002–5.11), respectively. The mean ratio of linezolid concentration in diseased bones/plasma was higher than that in nondiseased bones/plasma, and the difference was statistically significant (*t* = 2.55, *p* = 0.025). Table 2 shows the linezolid concentrations in different sample types of spinal lesions.

**Table 2:** Linezolid concentrations in different sample types of spinal lesions

Patient No.	Specimen collection time (min)^c^	Concentration (mg/L) of linezolid in:				Ratio^a^		
		Plasma	Nondiseased bone	Diseased bone		Nondiseased bone / plasma	Diseased bone / plasma	Diseased bone / nondiseased bone
1	139	2.16	0.96	1.63		0.44	0.75	1.70
2	150	9.45	4.05	5.39		0.43	0.57	1.33
3	180	3.44	1.01	2.13		0.29	0.62	2.11
4	220	3.29	0.65	1.87		0.20	0.57	2.88
5	140	12.03	1.96	3.50		0.16	0.29	1.79
6	105	9.05	0.63	3.22		0.07	0.36	5.11
7	200	8.231	2.025	2.030		0.246	0.247	1.002
Median	150	8.23	1.01	2.13		—^b^	—^b^	—^b^
Mean	—^b^	—^b^	—^b^	—^b^		0.26	0.49	2.27
SD	—^b^	—^b^	—^b^	—^b^		0.14	0.19	1.39

^a^Ratio of linezolid concentrations in the two samples

^b^No data

^c^Time from administration of preoperative dose of linezolid in the morning to sample collection during the operation

### Correlation analysis of linezolid concentrations in diseased bone and nondiseased bones

Pearson’s correlation analysis revealed the positive correlation of linezolid concentrations in the diseased and nondiseased bones (*r* = 0.810, *p* = 0.027).

## Discussion

Different TB drugs exhibit various penetrabilities in TB lesions, and spatial heterogeneous distributions can be observed when the same TB drug is used in distinct disease types of the same TB lesion [13–14]. A few studies have focused on the distribution of linezolid in TB lesions in patients with spinal TB. Our previous works showed the different spatial concentration distributions of linezolid in TB lesions in patients with spinal MDR-TB [11, 15], but they did not compare the distributions of linezolid in diseased and nondiseased bones. Therefore, we conducted this study to further elucidate the distribution of linezolid in diseased and nondiseased bones in patients with spinal TB. Pharmacodynamic and pharmacokinetic investigations of linezolid based on its spatial distribution in spinal TB lesions may help to solve the problems in its clinical use.

The reports on the simultaneous distribution of linezolid in both diseased and non-diseased tissues of only one organ are limited [12, 14]. Correspondingly, the distribution of linezolid in different tissues of diseased organs is unknown. A previous study reported that the median ratios of linezolid concentration in nondiseased lung tissue/plasma and diseased lung tissue/plasma of patients with drug-resistant TB were 0.41 and 0.49 respectively; moreover, linezolid showed better penetration in diseased lung tissues than in nondiseased lung tissues (0.41 vs. 0.49) [12]. The current research also unveiled the same phenomenon: linezolid exhibited a good penetration capability in diseased bones compared with nondiseased bones (0.49 vs. 0.26). Moreover, the linezolid concentration in diseased bones showed a positive correlation with that in nondiseased bones (*r* = 0.810, *p* = 0.027), which was also observed in a previous study [16]. These findings suggest that linezolid concentrations in different types of bones in spinal TB patients are correlated.

This research revealed the better penetration of linezolid in diseased bones than in nondiseased bones. This finding suggests that TB infection increases the penetration of linezolid into diseased bones. The possible reason may be the TB infection increasing the penetrating capability of linezolid to blood vessels and bone tissues in diseased bones. In this study, the mean ratio of linezolid concentration in diseased bone/nondiseased bones was 2.27. The high concentration distribution of linezolid in diseased bones is beneficial to the control of TB infection. This condition may also explain the good effect of linezolid on the treatment of spinal MDR-TB [17]. In this study, the ratio range of linezolid concentration in diseased/nondiseased bones was 1.002–5.11, which is consistent with those of a previous report [12]. The differences in the ratio of linezolid concentration in diseased/nondiseased bones of different patients may be due to the varying diseased bone structures and permeability of the vascular wall in different individuals infected by *M. tuberculosis*. As a result, linezolid exhibits varying penetrabilities in bone lesions in various patients.

In this study, the mean ratio of linezolid concentration in diseased bones/plasma was 0.49, which is consistent with those of previous reports (range: 0.42–0.53) [11, 18]. A past study reported that the mean ratio of linezolid concentration in degenerative bone/plasma was 0.3986 (range: 0.177–0.978) [19]. In another research, the penetration values of linezolid in sternal cancellous bone during coronary artery bypass grafting were 0.82 and 1.02 (AUC_2.5–24_ tissue (µg/h/ml)/AUC_2.5–24_ plasma (µg/h/ml)) [20]. In this study, the mean ratio of linezolid concentration in nondiseased bones/plasma was 0.26 (range: 0.07–0.44), which is lower than previously reported values [19, 20]. The mode of administration of linezolid used in previous studies was as follows: Linezolid 600 mg 12 hourly was given orally over the 48 h before operation and intravenously 1 h before induction of anaesthesia [19]; 600 mg linezolid was administered continuously over 30 min, starting 60 min prior to skin incision, and twelve hours after the first antibiotic administration of linezolid, a second dose of 600 mg was infused [20]. The differences in the penetrating capability of linezolid in these studies may be related to the various administration methods, sampling sites, and detection methods used. The specific reasons need to be further studied.

This study has several limitations. First, this research involved a small cohort of patients with spinal TB. Second, this work is a retrospective study with uncontrollable factors.

In summary, linezolid showed a higher concentration distribution in diseased bones than in nondiseased bones, and its concentrations in diseased and nondiseased bones were positively correlated.

## Data Availability

All data supporting the findings of this study are available from the corresponding authors upon reasonable request.

## Abbreviations

MDR: multidrug-resistant
HPLC: high-performance liquid chromatography
SD: standard deviation
BMI: body mass index
T: thoracic vertebra
L: lumbar vertebra
